# Spatio-temporal pattern formation of living organisms at the edge of chaos

**DOI:** 10.1093/ismejo/wraf050

**Published:** 2025-03-13

**Authors:** Johannes Werner, Hartmut Arndt

**Affiliations:** Department of General Ecology, Institute of Zoology, University of Cologne, Zülpicher Str. 47b, Cologne 50674, Germany; Department of General Ecology, Institute of Zoology, University of Cologne, Zülpicher Str. 47b, Cologne 50674, Germany

**Keywords:** self-organization, spatio-temporal pattern formation, microfluidic chip, *Tetrahymena pyriformis*, chaotic dynamics, ecological complexity, population dynamics, biodiversity

## Abstract

Understanding spatio-temporal dynamics is essential for predicting how populations fluctuate over time and space. Theoretical models have highlighted the ecological complexity of spatio-temporal dynamics, which can lead to the emergence of complex patterns, including nonlinear dynamics and chaotic behavior, important mechanisms for maintaining of biodiversity. However, these dynamics are difficult to observe experimentally due to a lack of temporal and spatial resolution. Here, we show that even a single-species system exhibits complex spatio-temporal patterns without external forcing where order and chaos coexist (edge of chaos). Automated analyses of experimental dynamics of cells of a ciliate on a microfluidic chip environment with 50 interconnected patches documented pattern formation, including chaos-like dynamics, using several analytical methods. Different initial conditions caused changes in patterns, revealing the complexity and principal unpredictability of self-organized pattern formation. A model containing the stochastic fluctuations of the experiment verified the deterministic nature of patterns. The results show the intrinsic complexity of ecological systems, challenging predictions in nature conservation. Our results bridge the gap between theoretical models and experimental observations, offering new insights into the fundamental nature of living systems and their spatio-temporal organization.

## Introduction

The process of spatio-temporal dynamics is inherently complex, driven by multiple interactions and nonlinear dynamics. Spatial heterogeneity is often attributed to differences in abiotic environmental factors, exemplified by the Moran effect—a synchrony caused by spatially correlated environmental influences observed in nature and in the lab [1–4]. However, major challenges in predicting spatial structures arises from individual behavior rather than spatial structures [5]. Also, self-organized spatial heterogeneity, driven by interactions between organisms, is rarely considered and has mostly been demonstrated in models [6, 7]. Accounting for these processes is important as they can enhance local diversity in metacommunities [7] and create spatial niches that further promote biodiversity [8]. Examples from literature include self-organized patterns in vegetation [9], plant-parasite interactions [10] and plankton–fish systems [11] and have been modeled in networks of discrete spatial patches [7, 12]. Self-organized pattern formation in ecological systems can be attributed to several mechanisms [13]. These include density-dependent movement, where individuals alter their behavior based on local population density [14, 15], and scale-dependent feedbacks, involving positive and negative interactions at different spatial scales [7, 9, 16]. These mechanisms provide a foundation for understanding self-organized patterns, but requirements and processes involved in their formation remain an active area of research.

Time-series analyses of distribution patterns can offer insights into the process of pattern formation as it unfolds over time [17]. Here, complex nonlinear dynamics can be observed, ranging from damped oscillations to stable limit cycles and deterministic chaotic dynamics [18, 19]. Deterministic chaotic dynamics are defined by aperiodic fluctuations sensitive to initial conditions. In experimental systems, it is difficult to distinguish chaotic from stochastic fluctuations [18, 20, 21] and few studies have considered chaotic dynamics under controlled environmental conditions [2, 22–26]. Even within individual species, complex behaviors without external forcing have been shown in models [19, 22, 27] and some experimental studies [22, 28], but chaos was still often expected to be an exception. After extensive simulation tests, a recent review showed that >30% of time-series data in a global database exhibited chaotic dynamics [20]. Methodological limitations have often led to misclassifications in the past [20]. As the causes of dynamics in biological systems are often not well understood [29, 30], detection of chaos relies heavily on mathematical models, which alone are insufficient to demonstrate the occurrence of chaos within the system itself. Due to inherent complexity of biological systems, theories suggest that dynamics often operate at a mixture of chaos and order, referred to the “edge of chaos” where complex systems can adapt and evolve most effectively [31–33]. Confirming whether chaotic and self-organized dynamics influence natural pattern formation could have fundamental consequences for understanding the enormous biodiversity, as these complex nonlinear dynamics can contribute to the stable coexistence of species and different genotypes [7, 8, 34]. To address this issue, a real-world system would be required in which extrinsic environmental factors are excluded, and all dynamics are based on intrinsic processes. Here, we show the complexity of spatio-temporal dynamics of a single species based on an experimental microfluidic chip in a controlled environmental system.

**Figure 1:**
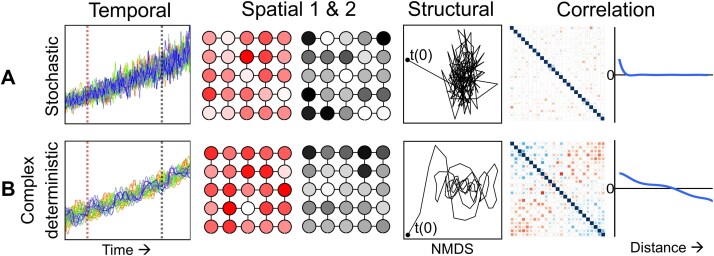
Hypotheses on the spatio-temporal distribution of single species within a network of equally resourced discrete spatial patches. (A) Stochastic hypothesis: Organisms are primarily randomly distributed and movement is density independent, characterized by insensitive initial conditions, lack of structure and correlations. This scenario was simulated using the same stochastic model as for the experiments (Material and Methods). (B) Complex deterministic hypothesis: Organisms follow a complex distribution process and movement is density dependent, which is mainly shaped by the initial conditions, characterized by a visible structure, partial correlation and possible complex dynamical behavior. For this scenario, a model was developed where the movement between chambers is governed by a nonlinear function of cumulative population differences, introducing density-dependent behavior. Both scenarios were simulated (supplementary text) using the same initial conditions exemplified by a theoretical network of 25 connected habitat patches, as visible in the spatial analysis. The temporal analysis (column 1) shows the abundance for each of the 25 chambers in different colors over time. The spatial analysis (columns 2 & 3) shows the distribution at two selected time points (spotted lines in corresponding graph of column 1). Structural analysis (NMDS with Bray Curtis; column 4) shows the structural distribution over time (first time point t(0)). Correlation analysis (columns 5 and 6) includes the pairwise spearman correlation coefficients among the 25 patches (column 5) and a plot of these coefficients against the minimum distance between patches (column 6).

For the spatio-temporal distribution of a single species in a network of different habitats with uniformly distributed resources, we hypothesize the existence of two possible ways of pattern generation: (i) Stochastic distribution: Density-independent distribution with stochastic fluctuations. (ii) Complex deterministic distribution: Density-dependent movement rates between patches (transition rates) leading to pattern formation. This would result in a complex distribution behavior of organisms such as chaos with changing variations and different visible patterns (see Fig. 1).

Because spatial distribution is not only a feature of movement but also a central life history trait [35], the high proportion of nonlinear interactions in nature [36, 37] could lead to complex distribution patterns even without external influences. Although the single-species system appears to be very simple, the complexity of the spatio-temporal dynamics reveals a system on the edge of chaos, highlighting the complexity of intrinsic mechanisms already present at the level of single-species.

## Materials and methods

### Microfluidic Chip

**Figure 2:**
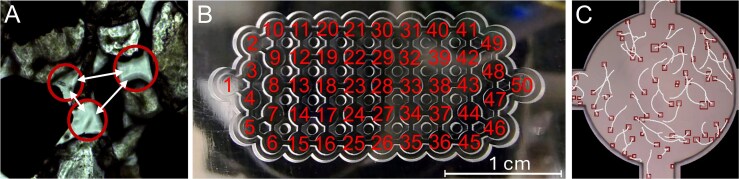
Experimental design of the microfluidic chip. (A) Microscopic image of ciliates from the interstitial region of a sandy beach (Helgoland Island, North Sea, Germany), serving as an ecological reference for distinct environmental patches, analogous to those in our microfluidic chip. (B) The microfluidic chip with all chambers numbered as used later in the presentation of results. (C) Video-view of a microfluidic chip habitat patch, highlighting ciliate cells with squares and their movement trajectories. For further details, see Methods section.

The microfluidic chip contains 50 interconnected patches, each 2 mm in diameter and 500 μm in height, making it suitable for studying a variety of unicellular organisms. With the exception of the patches far left and right each patch interfaces with three neighboring patches, ensuring a uniform connectivity across the chip. Constructed from Plexiglas, the chip is both reusable and easy to clean. The microfluidic chip is milled into a 3.5 mm thick PMMA (Plexiglas) plate, allowing for detailed inspection using an inverted microscope equipped with a video camera and an automated X-Y positioning system that allows to scan cells in each patch (Fig. 2B, C).

### Experiments

An axenic culture of the ciliate *T. pyriformis* (CCAP 1630/1 W) was obtained from the Culture Collection of Algae and Protozoa (Scotland, United Kingdom). The ciliates had an average size of approximately 85 μm × 22 μm. Prior to the experiment, cultures were adapted to the experimental temperature (15°C) for 24 hours. All 14 experiments were performed under constant conditions. The culture and PPY (Proteose Peptone Yeast Extract) medium were mixed until the respective abundance was reached and then used to fill the chip (120 μl). The chip was covered and sealed with grease (Borer glisseal N, Carl Roth, Karlsruhe, Germany) to minimize evaporation. After filling and sealing the chip, the experiment was immediately started. The abundance of each patch was determined sequentially, with the microscope table movement automated by three stepper motors (ST2818S1006-B, NEMA 11, Nanotec, Feldkirchen, Germany) and controlled with a self-programmed application running in the Python script language. This setup allowed the abundance of each patch to be measured every 6:20 minutes. For each patch, five seconds of video (with a resolution of 2048 × 2048 pixels and 35 frames per second) were recorded for abundance determination using a MikroLive 5MPplus camera (Microlive, Schifferstadt, Germany) with a Sony IMX264 sensor. A recorded timestep symbolizes a complete measurement cycle covering all 50 patches sequentially. For all experiments, 105 timesteps (12 hours of experimental time) were recorded. Different initial conditions between experiments were realized through a combination of varying inoculation densities (ranging from 1159 to 5869 cells) and stochastic deviations between patches.

### Measurement of abundance

The entire measurement and analysis process was fully automated. Over the course of 14 experiments, more than 100 hours of video footage were recorded, containing over 12 million frames. After capture, the videos were transferred to the High Performance Computing (HPC) system CHEOPS of the University of Cologne and analyzed with a custom-trained neural network based on the Ultralytics Yolov8 algorithm [38] (v. 8.0.227) algorithm and implemented with the PyTorch framework [39](v. 2.2.0.dev20230914 + cu121). As an additional step to reduce the number of false positive detections, the positions of all detections were stored and analyzed for movements. If a detected cell did not move during the 5-second video recording, it was excluded from the analysis. This was ensured by tracking the position of every detection. If a detection did not move beyond its initial position ±4 μm (approximately 5% of the average cell length), it was discarded. This led to a reliable analysis of the abundance data (*P* = .982, R = 0.974, mAP50 = 0.989, mAP50–95 = 0.879).

### Analysis of experimental data

Data analysis was performed using R (v.4.1.1, Vienna, Austria) (R Core Team 2021) and MATLAB version R2023b (MathWorks, Inc., MATLAB, Natick, 2023). All experimental data were analyzed (i) for their temporal dynamics (time series analysis) and (ii) for their spatial distribution using the timesteps.

The temporal dynamics of the system were analyzed by examining the abundance of each patch over time. One of the most commonly used indices for detecting chaotic dynamics is the Lyapunov exponent (LE), which measures the average rate of divergence between nearby points in the phase space [20, 21, 40]. Positive LE values indicate chaotic dynamics. The LE was estimated using two methods, the direct method after Rosenstein [21] and the indirect Jacobian matrix method [40]. The LE estimations, including estimates of the hyperparameters (embedding dimension and time lag), were done according to a previously published study [20]. Their findings demonstrated that the Jacobian LE method provides the most conservative estimates and performs best on shorter time series. As a further buffer against false positives, we followed their procedure that the minimum lower bound for LE had to be >0.01 for the time series to be classified as chaotic. Additionally, a sliding window approach using 50-timepoint-long time series was employed to determine local LE, capturing stationary data and obtaining information on whether the local stability changed throughout the experiment. This approach resulted in 55 measurements for each time series. Furthermore, the hyperparameter (θ) of the optimized model for the Jacobian LE was examined to verify that positive LE were not solely a reflection of non-stationary exponential growth [41]. When this parameter was non-zero, it indicated that a non-linear model performed best for prediction in the S-map framework. In such cases, we considered a positive LE as a reliable indicator of chaos. Positive LE alone (with θ = 0) were classified as a mixture of chaotic dynamics and exponential growth and were not included in the discussion.

For spatial analysis, each timepoint was analyzed regarding the autocorrelation using Moran’s I statistic, implemented with the R package “spdep” (v.1.3–1) [42]. *P* values were calculated through Monte-Carlo permutation simulations using the moran.mc() function, with 12 000 permutations to ensure reliable *P* values after correction. The algorithm utilized the network of connected patches to determine spatial relationships in abundance patterns.

Spatial correlations were calculated through pairwise Spearman rank tests on the time series data for each patch. In complex, nonlinear systems, correlations can fluctuate rapidly, causing problems in causal analysis [37]. To accurately determine the correlations, all abundance values were normalized by the total abundance at each timestep, thereby offsetting the influence of overall growth and minimize non-stationary effects. To control for false discovery rates (FDR), *P* values from the autocorrelation and correlation tests were adjusted using Holm’s method [43].

Using the Bray–Curtis dissimilarity index [44] on the abundance data across different time points, a PERMANOVA (permutational multivariate ANOVA) was performed to determine whether and how much of the variance could be attributed to time in the model. Additionally, a second dissimilarity matrix after Dynamic Time Warping (DTW), using the R package “dtw” [45, 46] was created. This matrix was used to perform a separate PERMANOVA to evaluate how much variance the position of each patch on the chip explained in the model. DTW, a robust distance measure for time series data, is commonly utilized for classification tasks [42, 46]. The *R*^2^ value was used to quantify the proportion of variance explained by time and position, respectively. Both the Bray–Curtis dissimilarity index and the PERMANOVA analyses were carried out using the R package “vegan” (v.2.6–6.1) [47].

To ensure the robustness of the findings, all analysis methods were also applied to a mathematical “null” model to determine whether the observed patterns were deterministic or could be explained by stochastic variation.

### Model

To test for deterministic aspects of the experimental data, a mathematical null model was developed based on experimental parameters but containing only random fluctuations. To determine the model’s movement (transitions) between patches, three separate experiments were conducted using cells of *T. pyriformis* under the same conditions as the main experiment. In each experiment, one patch was monitored with over 500 time points, with cell counts taken every two minutes via automated abundance measurement (see above). By tracking the movement of cells, the influx, and efflux rates for the patch were quantified. The analysis showed a significant flux of cells between patches, with a transition rate of 60% ± 15% (mean ± SD) per minute, with the distribution of transitions between patches following an approximately normal distribution (Fig. S7). These findings were consistent across all three calibration experiments, each using different patches.

Alongside the growth rate (g) obtained from the experiments, the model’s framework for the stochastic fluctuations could be determined. This model consists of 50 coupled ordinary differential equations (ODEs), where each equation describes the abundance within a patch (measured in cells per patch). Transition rates were randomly selected from a set of values following a normal distribution with μ as the expected value of 60% and σ as the standard deviation (15%). Transition rates between connected patches were stored for each timestep in a 50x50 matrix, denoted as matrix A_t_. In this matrix, each element a_i,k_ represents the transition from patch i to patch k. The mathematical ODE system was therefore described as follows:


$$ {A}_t=\left(\begin{array}{@{}ccc@{}}{a}_{11}& \cdots & {a}_{k1}\\{}\vdots & \ddots & \vdots \\{}{a}_{i1}& \cdots & {a}_{ik}\end{array}\right)\kern-3pt \mid\kern-1pt {a}_{ik}\, \textrm{normal}\, \textrm{distributed}\, \textrm{after}\, \mu\, \textrm{and}\, \sigma\, \textrm{for}\, \textrm{all}\, t$$

$$\kern-8.5pc \frac{d{y}_i}{dt}=g\ast{y}_i-{y}_i\ast \sum_{k=1}^{50}\left({a}_{ik}\right)+\sum_{k=1}^{50}\left({a}_{ki}\ast{y}_k\right) $$

For the growth rate, values following normal distribution with g = 4.51 ± 0.76 d^−1^ were used (see Source Data Model). The calculations were performed using MATLAB version R2023b (MathWorks, Inc., MATLAB, Natick, 2023).

To compare the stochastic model with the experimental data, 1000 model outputs were generated using the 14 different initial conditions from the experiment. Statistical analysis was performed to compare the model and the experiment using the Wilcoxon rank-sum test, which is robust against unequal sample sizes (n = 14 for the experiment and n = 1000 for the model). To account for multiple comparisons and reduce the FDR, *P* values were adjusted using Holm’s method [43].

## Results

### Experimental spatio-temporal dynamics on a microfluidic chip

**Figure 3:**
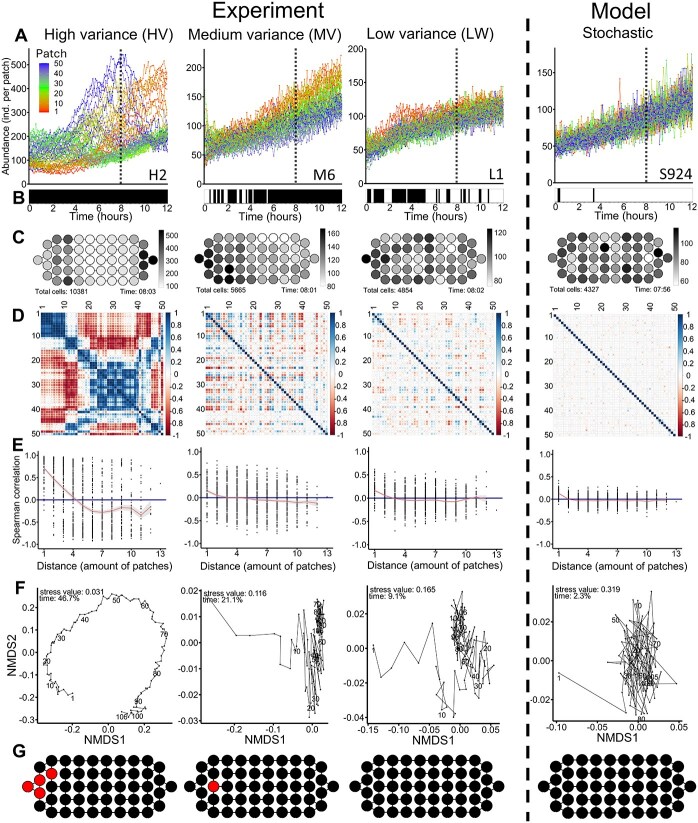
Analysis of spatio-temporal dynamics of cells of *Tetrahymena* across experimental and model data. (A) Temporal abundance trajectories across all 50 patches in a single experiment. Colours represent the different patch positions, ranging from patch 1 to patch 50 (the sequence of labelling follows the rainbow colours). The patch numbers correspond to those shown in Fig. 2. Among the 14 independent experiments, three significantly different categories with HV (n = 3), MV (n = 8) and LV (n = 3) were separated. for each category one example is shown. The number in the right lower corner of the graphs resemble the number of the experiment as indicated in Figs. S1–S7. For the model simulations different initial abundances were used, here one of the experiments LV (L2) is shown. (B) Autocorrelation analysis for each time step based on Moran’s I. Positive autocorrelation is indicated in turquoise, whereas red indicates the absence of autocorrelation. Statistical significance was assessed using Monte Carlo permutation simulations, with an FDR adjusted; *P* < 0.05 considered significant. (C) Spatial distribution of cell abundance after 8 hours, represented schematically on the experimental chip. Abundance is displayed using a grayscale gradient, with white indicating low abundance and black indicating high abundance. (D) Pairwise spearman rank correlation coefficients for the abundances across all 50 patches. The coefficients are color-coded as visible in the legend. (E) Spearman correlation coefficients plotted against the minimum distance between chambers, with direct neighbours having a distance of 1. The smoothed line represents the mean ± s.e.m. (F) Non-metric multidimensional scaling (NMDS) plot based on Bray–Curtis dissimilarity, using abundance data from all patches. Points are connected sequentially according to their time points. The stress value and PERMANOVA *R*^2^ for the time factor are provided. (G) Chaos classification based on the Jacobian LE method. The schematic representation of the chip highlights time series classified as chaotic (red) and non-chaotic (black).

**Figure 4:**
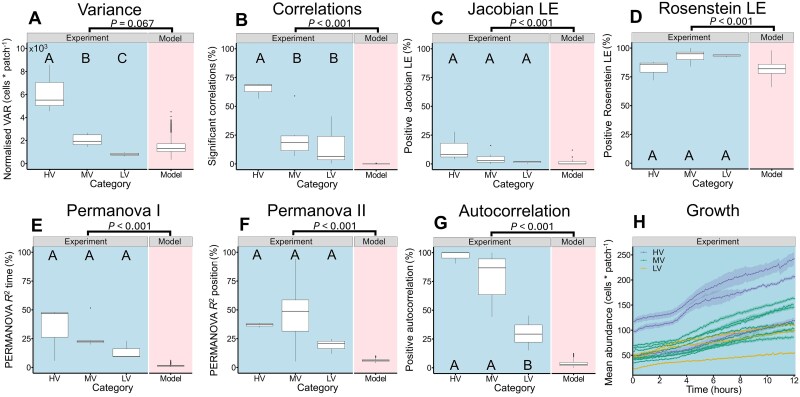
Comparative analysis of *Tetrahymena* spatio-temporal dynamics. (A-G) Comparative analysis of HV (n = 3), MV (n = 8), and LV (n = 3) experimental categories. Statistical significance was assessed using one-way ANOVA followed by Tukey’s post-hoc test, with *P* < 0.05 considered significant. Additionally, experimental data (n = 14) were compared with the stochastic model (n = 1000) using a two-sided Wilcoxon rank-sum test, with significance determined by FDR-adjusted *P* < 0.05. (A) Normalized variance (variance divided by total abundance per timestep). (B) Percentage of significant correlations. (C) Percentage positive Jacobian LE. (D) Percentage of positive Rosenstein LE. (E) Percentage of explained variance in PERMANOVA for the factor time. (F) Percentage of explained variance in PERMANOVA for the factor position. (G) Percentage of positive autocorrelations. (H) Mean abundance of the different experiments (n = 14), with colours representing HV, MV, and LV classification (mean ± s.e.m.). All experiments were monotonically increasing (FDR-adjusted *P* < 0.001, Spearman’s rho).

The automated setup in the well-controlled laboratory system reliably measured the abundance of organisms in each patch at intervals of a few minutes. The monotonic trend in total growth (Figs. 3A, 4H), combined with the high precision and recall values of our model (see Methods section), indicated low observational errors and minimal noise. Despite equal and constant external conditions in each patch, experiments showed a complex and diverse dispersal of cells and distribution patterns. Cells of *Tetrahymena* were able to spread throughout the microfluidic chip and all experiments showed a positive growth rate (4.51 ± 0.76 d^−1^). Due to the positive growth, all-time series were non-stationary. Although the total abundance increased monotonically in the experiments (Figs. 3A, 4H), the abundances within the patches were highly variable. All experiments were inoculated with different starting cell concentrations from 1159 to 5869 cells (9.658–48.908 cells/ml, a range of protist abundances we found in the interstitial of North Sea sediments) to obtain a range of different conditions. Based on the normalized variance of abundances, the 14 independent experiments were classified into three significantly different categories with high-variance (HV), medium-variance (MV), and low-variance (LV) (Figs. 3, 4A). HV experiments had significantly higher starting abundances than MV and LV experiments (*P* = .030 and *P* = .039 respectively, Tukey-HSD). In addition, these three categories showed visibly different distribution phenomena, as the high-variation experiments showed clear peaks in patch abundances, the medium-variation experiments showed global patterns, and the low-variation experiments showed no clear pattern (Fig. 3A, Fig. S1). The continuous growth of cells indicates that they were not subject to any limiting conditions (Fig. 4H).

### Spatio-temporal patterns at the edge of chaos

Positive spatial autocorrelation, a hallmark of patchy distribution [1], was at least temporarily detected in all experiments (Moran’s I) (Fig. 3B–C, Figs. S1 and S2). HV and MV experiments showed the highest rate of positive autocorrelation (Fig. 4G). Negative autocorrelation was not observed in any of the experiments.

The pairwise Spearman correlations showed distinct patterns of correlation clusters, with no single pattern being fully replicated (Fig. 3D, Fig. S3). These patterns included a mixture of positive and negative correlations. The HV experiments showed particularly complex correlation patterns with the highest number of correlations (Figs. 3D, 4B). Correlations across distances provide information about the size of clusters and connectivity. With one exception, all experiments showed on average positive correlations between immediate neighbors (distance = 1), whereby this correlation generally decreasing up to a distance of 5. Beyond this range (distances 6–13), the correlation coefficients exhibited different trends, including increases, decreases, or constancy. LV experiments displayed the least variation in correlation with distance (Fig. 3E, Fig. S4).

**Figure 5:**
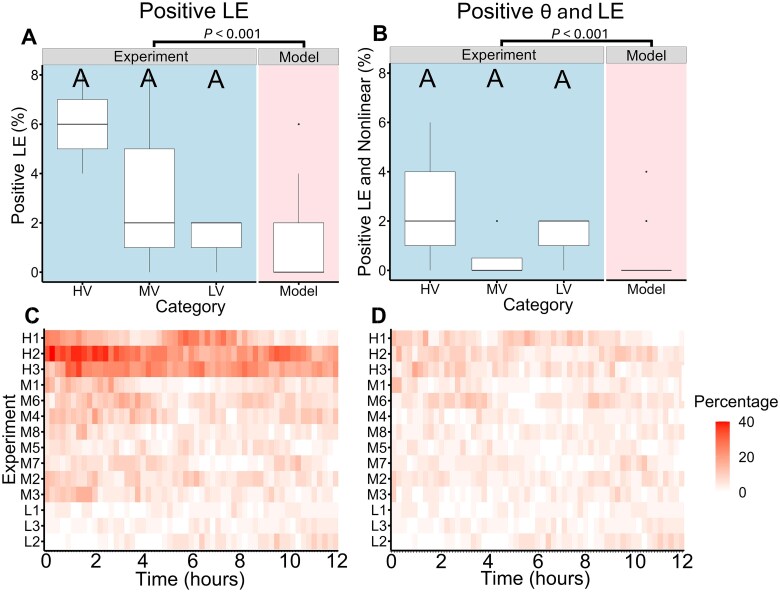
Comparison of linear and nonlinear Jacobian chaos and local stability classifications in *Tetrahymena* spatio-temporal dynamics. Chaotic classifications were based on the Jacobian LE method and nonlinearity estimates based on local weighting parameter θ [41]. Only when both parameters were positive, the dynamics were considered chaotic. (A–B) Comparative analysis of HV (n = 3), MV (n = 8), and LV (n = 3) experimental categories. Statistical significance was assessed using one-way ANOVA followed by Tukey’s post-hoc test, with *P* < 0.05 considered significant. Additionally, all experimental data (n = 14) were compared with the stochastic model (n = 1000) using a two-sided Wilcoxon rank-sum test, with significance determined by FDR-adjusted *P* < 0.05. (A) Percentage of time series with positive Jacobian LE. (B) Percentage of time series with positive Jacobian LE and positive θ values classified as nonlinear chaotic. C-D, Heatmaps showing the proportion of local instability estimates based on an analysis of each experiment using a sliding window approach with a length of 50 timepoints. With H1-H3 high-variance, M1-M7 medium-variance and L1-L3 LV experiments. The experiments in each category are ordered from top (high) to bottom (low) in their starting abundance. The color represents the percentage of chaos classifications either by the Jacobian LE (C) or by both Jacobian LE and θ (D), ranging from low to high.

Chaotic dynamics were analyzed using two distinct detection methods across the series of experiments. Changes of the abundance in individual patches over time (time series) were investigated. The Jacobian LE method detected at least one positive LE in 11 out of 14 experiments, with an average classification rate of 5.9 ± 7.7% (Fig. 3G, Fig. S6), indicating a mixture of chaotic dynamics and exponential growth. Excluding time series classified as linear reduced the percentage of positive LE to 1.2 ± 1.7% of the time series classified as chaotic. The direct LE determination using the Rosenstein method classified an average of 90.7 ± 7.3% of the cases with a positive LE exponent. No significant difference was found between the three categories for both chaos detections methods (Fig. 4C–D). The local LE (sliding-window approach) revealed a localized stability measurement, with all experiments exhibiting positive LE at some timepoints. Overall, 8.1 ± 6.7% of the time series exhibited a positive LE. Under nonlinear conditions, this proportion decreased to 4.8 ± 1.6% (Fig. 5C, D). Even under nonlinear conditions, HV experiments showed a significantly higher proportion of positive LE values compared to MV and LV experiments (*P* = .002 and *P* < 0.001, respectively, Tukey-HSD), with 6.2 ± 0.1% of time series classified as locally instable. In both methods the Jacobian LE were on average slightly negative, whereas the localized exponents were exhibiting a larger range of exponents (Table S1).The experiments revealed intricate spatio-temporal patterns that can be characterized as a mixture of chaos and order, often referred to as the edge of chaos [31].

### Unpredictability of distribution patterns

The PERMANOVA results indicated that, on average, 38.6 ± 23.1% of the variance was attributable to the patch position, with the chip length explaining more variance than the width (Fig. 2) (*P* = .031, Welch’s t-test). The second PERMANOVA analysis, focusing on the distribution patterns over time, revealed that 24.9 ± 14.3% of the variance was explained by time-related factors. In both models, the experimental category did not significantly influence the proportion of explained variance (Fig. 4E, F). Non-metric multidimensional scaling (NMDS) plots of Bray-Curtis distances (Fig. 3F, Fig. S5) visually represented the distributional changes over time, with points in close proximity indicating high similarity. For LV and MV experiments, the highest spatial changes occurred within the first 10–20 timepoints, highlighting a transient phase during which cells established their distribution patterns. In contrast, HV experiments exhibited more continuous spatial changes.

### Deterministic nature of distribution patterns

Statistical comparisons revealed no significant differences between the model and the experimental values in terms of total variance (FDR-adjusted *P* = .248, two-sided Wilcoxon rank-sum test), mean values and total abundances (FDR-adjusted *P* = .749, two-sided Wilcoxon rank-sum test) and normalized variance (Fig. 4A). These findings indicate that the basic framework of the model fits to our experiment. The model had significantly lower distribution parameters for the number of positive autocorrelations, explained variance by time and position and significant correlations (Fig. 3D–F, Fig. 4B, E–G). Additionally, chaos detection methods, including the Jacobian LE (0.8 ± 0.01%) and the direct Rosenstein LE (82.0 ± 5.4%), indicated significantly less positive LE in the model compared to the experiment (Fig. 4C, D). Even under nonlinear conditions, the proportion of positive Jacobian LE in the experiments was significantly higher than in the model (Fig. 5B), with the model showing falsely positive chaos in 0.2 ± 0.7%. Overall, the model did not exhibit the huge variability which was observed in the experiment (Fig. 3).

## Discussion

The experimental findings are in line with the discourse on the edge of chaos, which suggests that biological systems often operate near a critical regime where order and chaos coexist next to each other [31–33], which we were able to show here in a controlled experimental system with a protist population. Our study demonstrates this by revealing both chaotic dynamics (indicated by nonlinear positive Lyapunov exponents) and ordered patterns (shown by spatial self-organized patterns indicated by positive autocorrelation, PERMANOVA and correlations). Self-organized patterns, as observed in our experiments, may be a key mechanism driving living systems toward this edge of chaos [48].

The observed self-organized patterns were generated by active cell movement between patches, as no other factors were altered throughout the experiments. Understanding the individual behavior of individual specimens is highly important, as it might explain the spatial distribution on large scales better than the spatial structure of the landscape [5]. There are several mechanisms known which can induce spatial pattern formation [13], however self-organization in a constant environment was frequently attributed to scale-dependent feedback [7, 9] or density-dependent movement [14, 15]. Self-organization due to scale-dependent feedback could arise from different various processes. Potential contributors to pattern formation include pheromone production [49], net inflow of nutrients from proximate patches [7], accumulation of metabolic byproducts [50], and minor variations in oxygen concentrations [51]. The high abundances in certain patches in high variance experiments (Figs. 3, S1) could be additionally explained by density-dependent movement. High abundances connected with lower movement rates could lead to a cascade of increasing abundances. However, the subsequent decline in abundance after reaching a peak value indicates the involvement of additional regulatory mechanisms. These inherently nonlinear processes likely contribute to the complex dynamics observed. For example, nutrient depletion in densely populated patches might trigger dispersal, whereas nutrient-rich patches could attract more cells. Maybe future studies could experimentally verify these mechanisms through targeted manipulations of nutrient gradients or by tracking labelled cells to observe individual movement patterns in response to local density changes.

The 14 repeated experiments exhibited significant differences in patterns and dynamics (Figs. 3, 4). The three categories distinguished in low, medium, and high variance illustrate the observed diversity. In the LV experiments, the differences from the random model were minimal (Figs. 3, 4), suggesting that the observed patterns could be partly attributed to stochastic fluctuations. In contrast, the MV and HV experiments demonstrated clear and distinct pattern formation (Fig. 3). The starting abundance could play an important role here, as HV experiments had significantly higher starting values and exhibited significantly higher local instability under the sliding-window approach (Fig. 5C, D). Also, the stochastic initial distribution of cells across patches may have influenced the pattern formation, further highlighting the sensitivity of the system to initial conditions. By comparing the results to the random model, which exhibited significantly lower spatial autocorrelation, visible pattern formation, and overall correlation (Figs. 3, 4), we ruled out stochastic processes as the primary driver of pattern formation. Even with a high speed of movement of *Tetrahymena* cells of about 500 μm/s [52] which could result in a crossing time of the complete chamber in about 65 seconds, HV experiments showed clear peaks in specific patches and MV experiments showed patterns on a larger spatial scale and continuous wave-like distribution patterns (Fig. 3, Fig. S1). However, still over 60% of the variance remains unexplained in both PERMANOVA models, indicating substantial unknown effects and interactions, as well as poor predictability.

The dynamics observed in our experiments are transient and non-stationary over the observation period. This non-stationarity could potentially affect the interpretation of our chaos detection methods, which typically assume stationary dynamics [18, 20, 53]. To reduce this effect, local instability measurements with partially stationary data and nonlinear classifications (hyperparameter θ, [41]) were performed. Together with the nonlinear determination, the Jacobian LE method proves to be effective for classifying our data. Even when assuming significant observational errors, this method does not artificially inflate the frequency of detected chaos [20]. Whereas precise estimates of the LE require longer time series of 10^4^ timepoints or more, the sign of the exponent can be calculated with fewer timepoints [20]. This is the reason why we focused only on the qualitative classification of chaotic dynamics. The comparison with the stochastic model confirms our findings as the model had significantly lower chaotic classifications (Figs. 4, 5). In contrast, the direct LE method proposed by Rosenstein is unable to distinguish between divergence caused by chaos and that caused by noise [20]. As a result, this method likely overestimates the classification of chaotic dynamics, despite the low noise levels suggested by the highly controlled environment and accurate automatic detection. The chaos decision tree [53] offers a straightforward approach to classifying chaotic dynamics without requiring model parameter calibration or selection. However, this method was validated on time series with a minimum length of 1000 points for which it showed a very high accuracy but showed a reduced accuracy in detecting chaotic dynamics in shorter time series [20, 53, 54]. Consequently, the chaos decision tree was not suitable to estimate the presence of chaotic dynamics in our studies. Also, additional methods used in other studies [20] such as permutation entropy, recurrence quantification analysis and horizontal visibility graph require case-specific calibrations, which are not possible with the limited number of independent experimental time series. If a system is at the edge of chaos, not all processes need to be chaotic to generate unpredictability of the system [32, 33]. The local instability measurement (Fig. 5) visualizes the change of stability classifications over time, indicating that even if complete time series are not classified as chaotic, local dynamics can be instable. This is a typical characteristic for systems at the edge of chaos [55]. If individual time series within an experiment are chaotic, they will also influence the neighboring patches. Therefore, it has to be assumed that the whole system shows some degree of chaotic behavior, with the exception of individual patches. The exact strength or amount of chaotic dynamics is not necessary for this classification.

Our findings may have important implications for understanding biodiversity and ecosystem functioning. Previous studies have shown that spatio-temporal dynamics and fluctuating abundances contribute to stable species coexistence [7, 8], particularly in the presence of chaotic dynamics [23, 34, 56]. Chaotic dynamics have been observed in experimental multi-species systems without external triggers, suggesting that interactions between species could act as a main driver of chaos [23–25]. However, experimental evidence for single-species chaotic dynamics without external triggers are poorly studied [22, 28]. A mixture of chaotic and ordered dynamics has been observed only in a few field studies, such as voles in northern Fennoscandia [57], measles epidemics of large cities [58] and a cyclic rock colonization by barnacles and crustose algae [55]. But here we report this phenomenon for a constant environment and in a single-species system. It is therefore important to understand how systems at the edge of chaos function and what conditions are necessary for their emergence. Our experiment demonstrates that neither external triggers nor interspecies interactions are necessary to observe dynamics at the edge of chaos, as these complex behaviors are present even at the single-species level. The observed complexity in a seemingly simple system suggests that these behaviors may be more widespread than currently thought, potentially occurring across various scales of biological systems.

Our results confirm the hypothesis that populations of individual species exhibit complex nonlinear dynamics in time and space, leading to unforeseeable patterns (Fig. 1C). As our experimental system was well controlled, extrinsic factors were excluded, and still complex dynamics were visible. The system of different habitat patches can be applied to a variety of different ecosystems (Fig. 2A), e.g. ponds or lakes interconnected by dispersal [59, 60], soil pore spaces forming a complex network of small interconnected spaces [61], or marine snow in the oceans [62].

The diversity of patterns observed in our study (Figs. 3, 4, S1–S7) exemplifies the intricate complexity of the system, demonstrating how the complex dynamics of individual species vary across distinct habitat patches. These complex spatio-temporal dynamics may not be identifiable in non-spatial analyses. Furthermore, the order and self-organization demonstrates the system’s characteristics as one operating at the edge of chaos. Starting from approximately the same initial conditions, the experiments demonstrate the potential for chaotic dynamics in simple ecological systems. Identifying chaotic processes and differentiating them from stochasticity is important as it increases the predictability of systems, as chaotic processes are deterministic. This study provides insights into the complexity of single-species spatial dynamics, nevertheless further research is needed to determine the prevalence of such dynamics in natural ecosystems. This study not only bridges the gap between theoretical models and experimental observations but also provides new insights into the fundamental nature of living systems and their spatio-temporal organization.

## Supplementary Material

Werner_Arndt_Supplement_accepted_wraf050

Werner_Arndt_Table_S1_LyapunovMetrics_wraf050

S1_Model_file_Werner_Arndt_wraf050

## Data Availability

All data generated or analyzed during this study are included in the article and/or supporting information. Raw data are available from authors on request.

## Code availability statement

The code of the mathematical model is given as a Supplementary Information.
