# Supplementary material for: Spatio-temporal pattern formation of living organisms at the edge of chaos
Source: ISME J. 2025 Mar 13;19(1):wraf050. doi: 10.1093/ismejo/wraf050 (PMC11964086; doi:10.1093/ismejo/wraf050)
Supplement: Werner_Arndt_Supplement_accepted_wraf050 [file werner_arndt_supplement_accepted_wraf050.docx]

Supplementary Materials for

Spatio-temporal pattern formation of living organisms at the edge of chaos

Johannes Werner^a^, Hartmut Arndt^a^*

^a^Department of General Ecology, Institute of Zoology, University of Cologne, Zülpicher Str. 47b, Cologne (Köln), 50674, Germany

*Corresponding author: [Hartmut.Arndt@uni-koeln.de](mailto:Hartmut.Arndt@uni-koeln.de)

This file includes:

Additional Figures S1 to S7

#
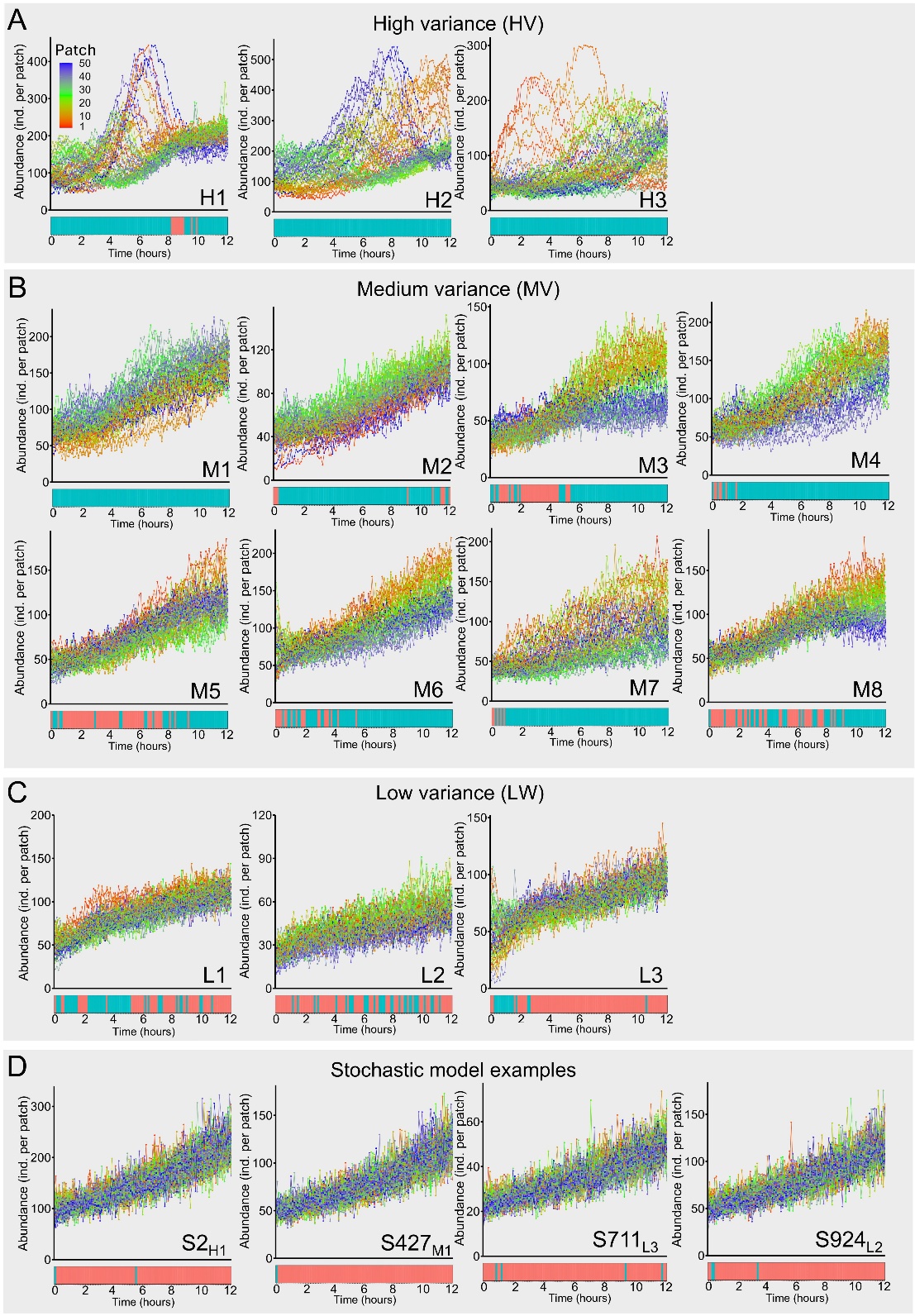


**Fig. S1. Abundance and autocorrelation across model and experimental data. A-D,** Temporal abundance trajectories across all 50 patches in a single experiment. Colours represent the different patch positions, ranging from patch 1 (red) to patch 50 (dark blue)(the sequence of labelling follows the rainbow colours). In the right lower corner of each graph, the number of the experiment is indicated which corresponds to the experiments shown in Fig. 1. The patch numbers correspond to those shown in Fig. 2. Below each graph, autocorrelation analysis for each time step based on Moran’s I is shown. Positive autocorrelation is indicated in turquoise, while red indicates the absence of autocorrelation. Statistical significance was assessed using Monte Carlo permutation simulations, with a false discovery rate (FDR) adjusted; P < 0.05 considered significant. Experiments were categorised corresponding their normalised variance. **A**, High-variance experiments (n = 3). **B**, Medium-variance experiments (n = 8). **C**, Low-variance experiments (n = 3). **D**, Random selection of four model simulations with different initial abundances corresponding to one of the experiments as indicated by the name in the right lower corner of each graph.


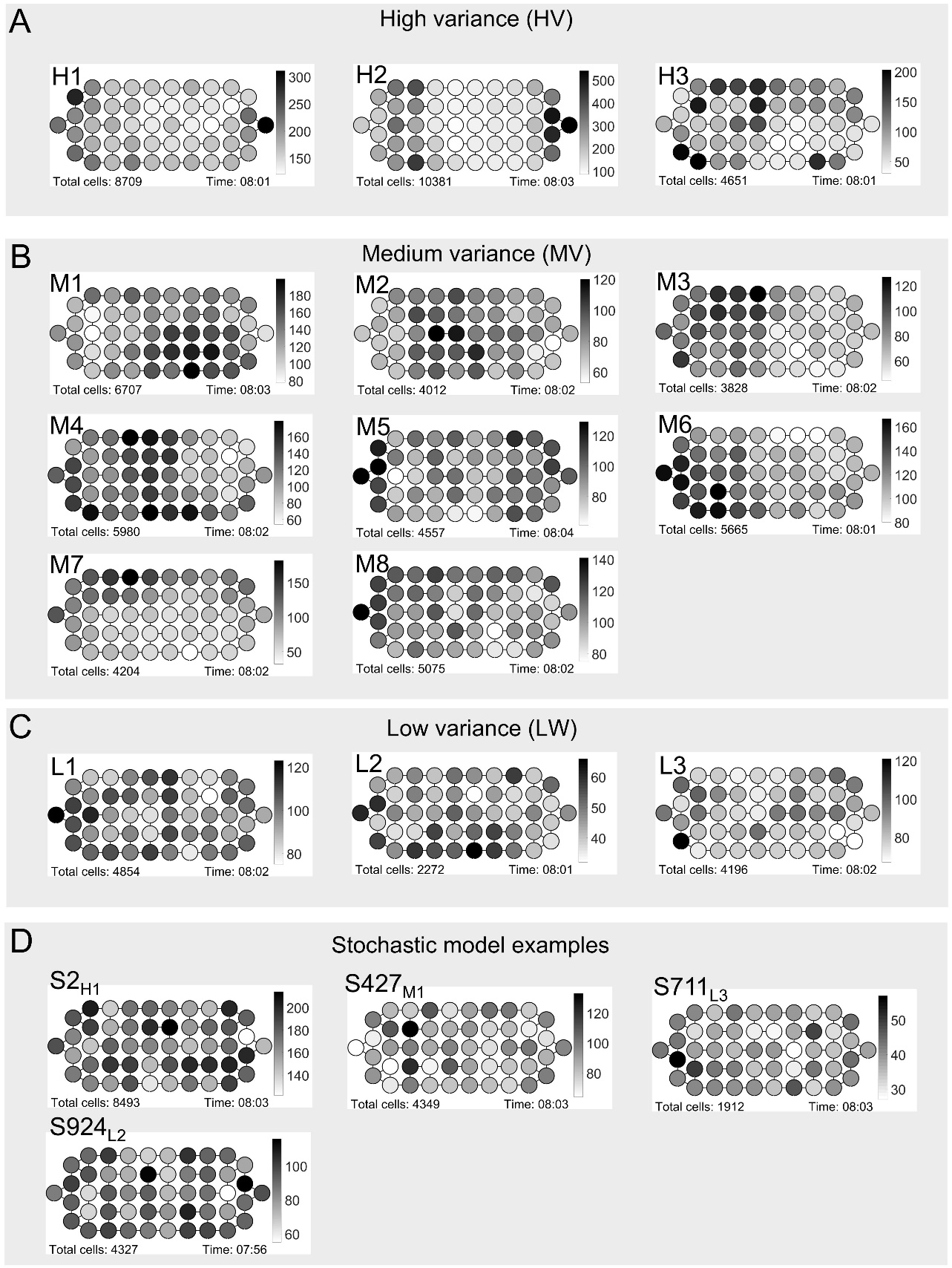


**Fig. S2. Spatial distribution of cell abundances across experimental and model data on the chip after eight hours. A**, High-variance experiments (n = 3). **B**, Medium-variance experiments (n = 8). **C**, Low-variance experiments (n = 3). **D**, Random selection of four model simulations with different initial abundances corresponding to one of the experiments as indicated by the name. Abundance value is indicated using a grayscale gradient, with white indicating low abundance and black indicating high abundance.


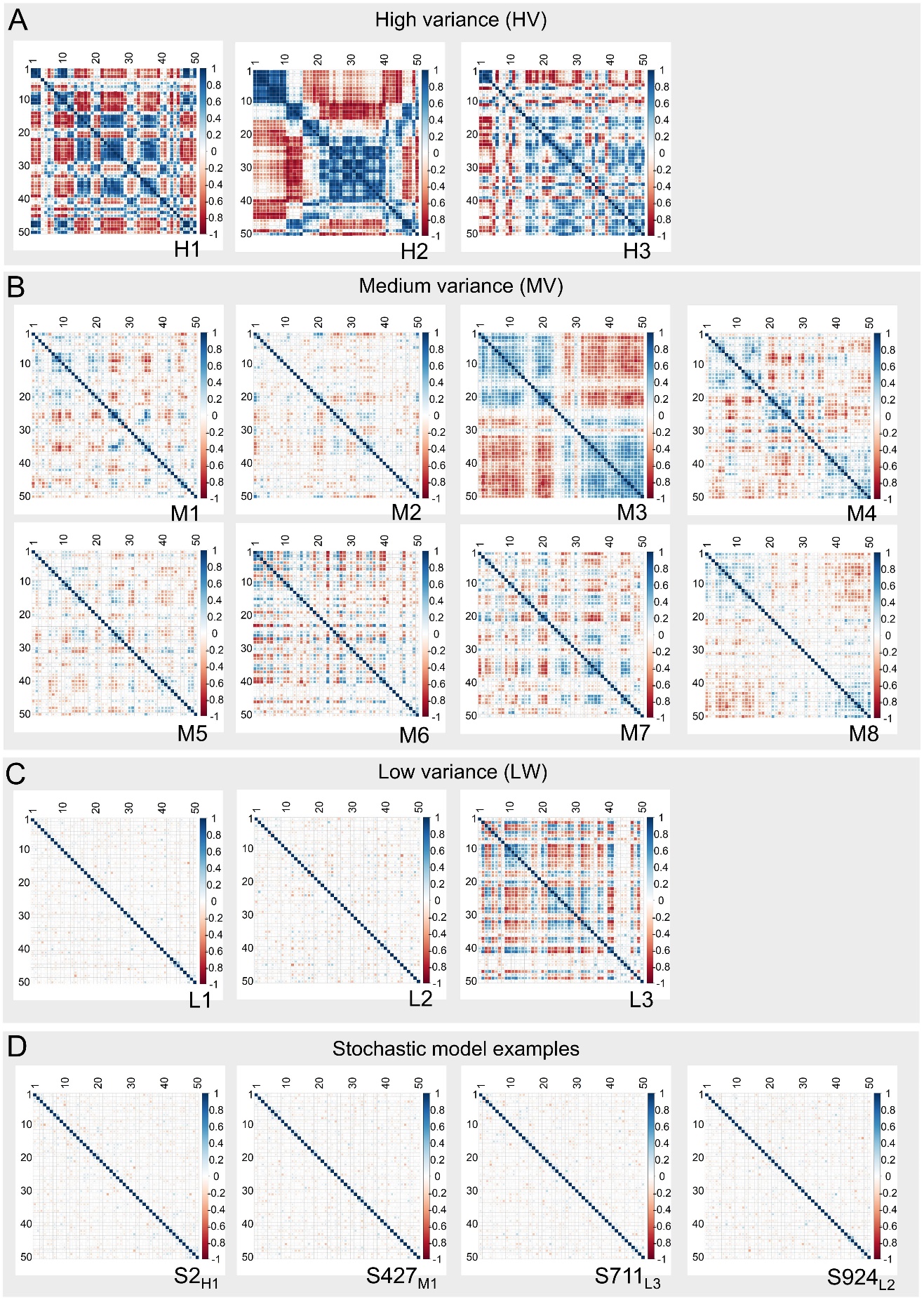


**Fig. S3. Results of the determination of correlation across experimental and model data.** Pairwise Spearman rank correlation coefficients are given for the abundances across all 50 patches. The coefficients are color-coded, with blue representing positive correlations and red representing negative correlations. **A**, High-variance experiments (n = 3). **B**, Medium-variance experiments (n = 8). **C**, Low-variance experiments (n = 3). **D**, Random selection of four model simulations with different initial abundances corresponding to one of the experiments as indicated by the name.


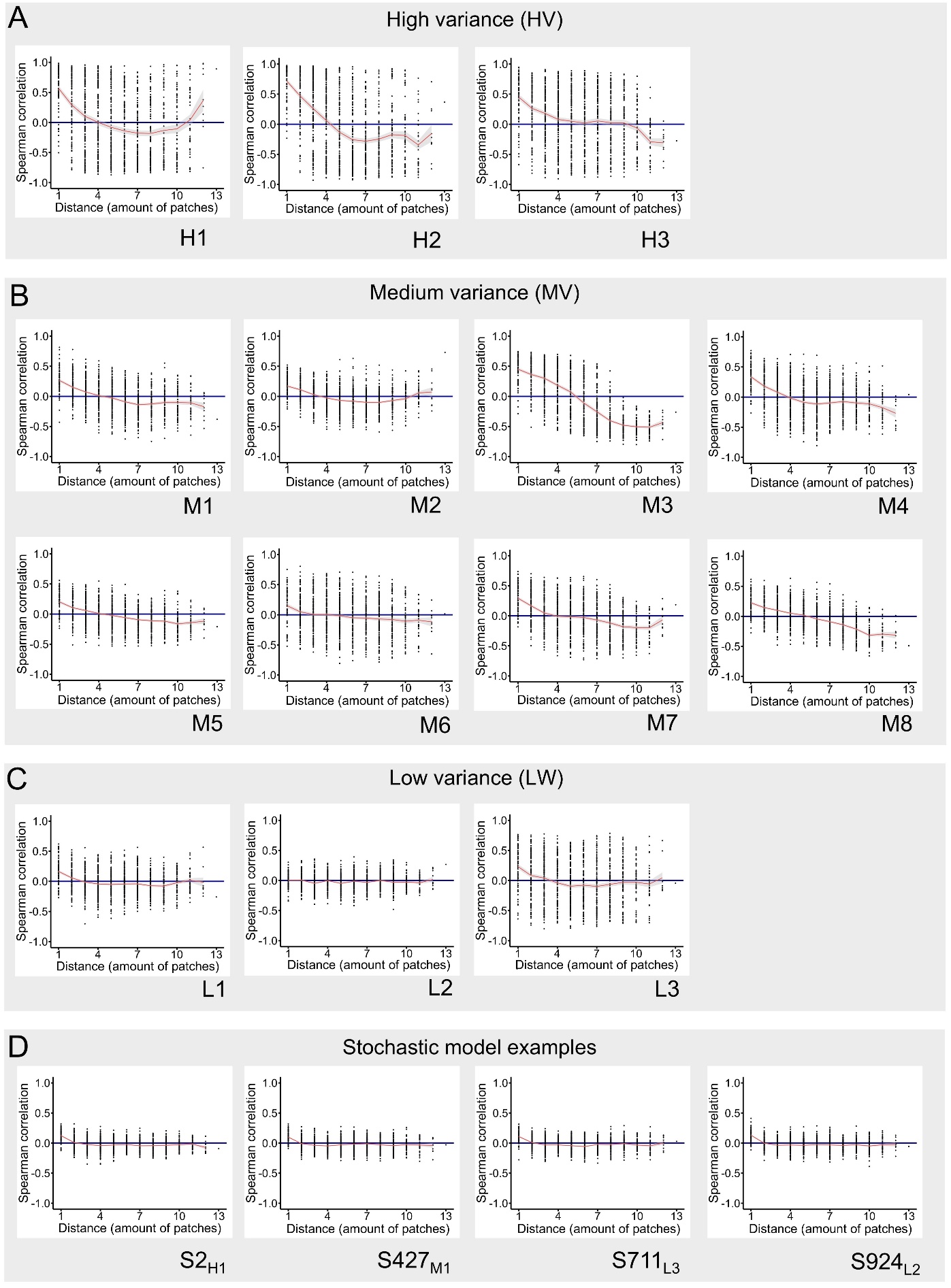


**Fig. S4. Spearman correlation coefficients of abundances plotted against the minimum distance between patches across experimental and model data.** Direct neighbours were considered having a distance of 1. The red line represents the mean ± s.e.m. **A**, High-variance experiments (n = 3). **B**, Medium-variance experiments (n = 8). **C**, Low-variance experiments (n = 3). **D**, Random selection of four model simulations with different initial abundances corresponding to one of the experiments as indicated by the name.


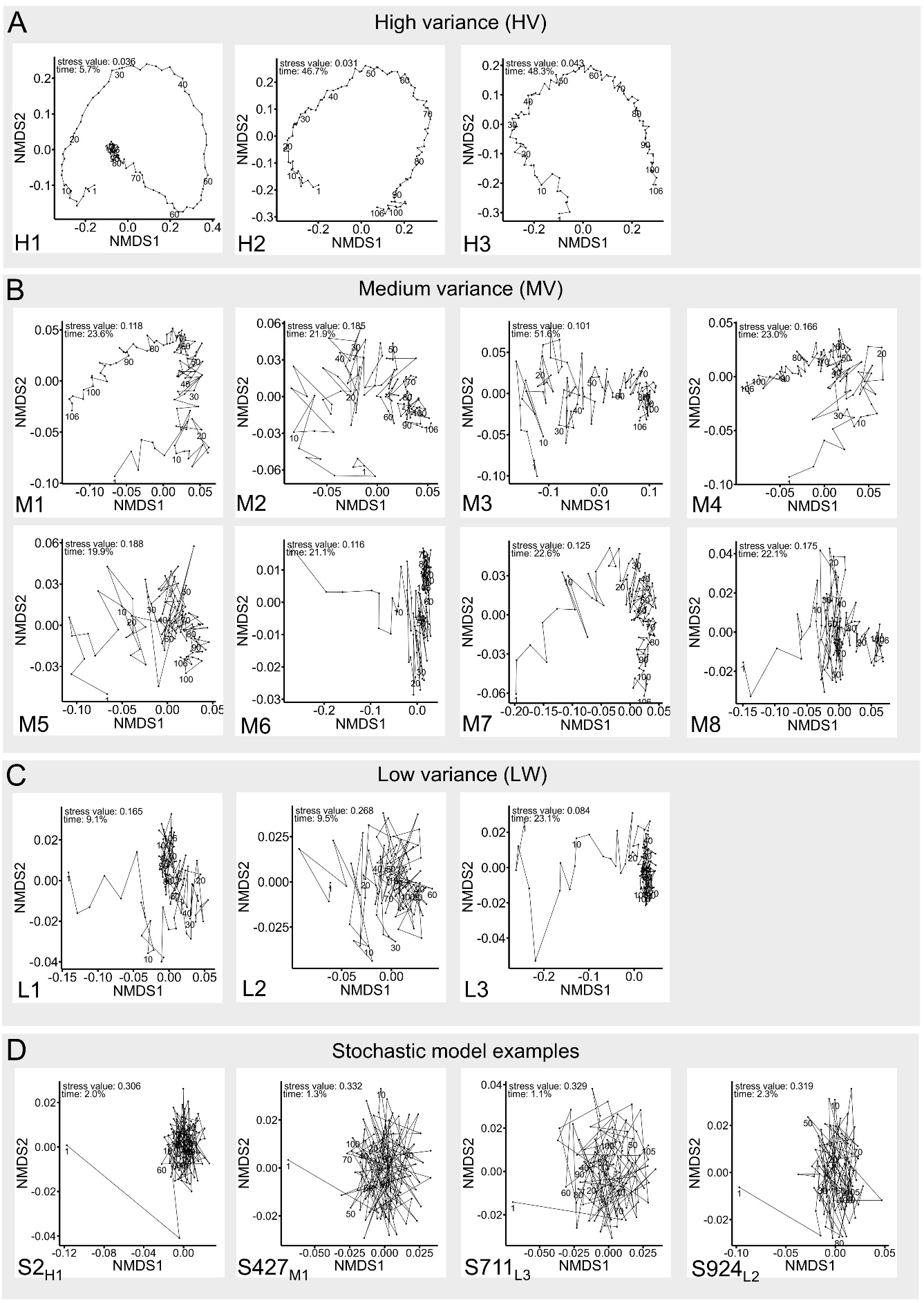


**Fig. S5. Non-metric multidimensional scaling (NMDS) plots based on Bray-Curtis dissimilarity across experimental and model data.** Points are connected sequentially according to their time points. The stress value and PERMANOVA R² for the time factor are provided in the upper left corner of each graph**. A**, High-variance experiments (n = 3). **B**, Medium-variance experiments (n = 8). **C**, Low-variance experiments (n = 3). **D**, Random selection of four model simulations with different initial abundances corresponding to one of the experiments as indicated by the name.


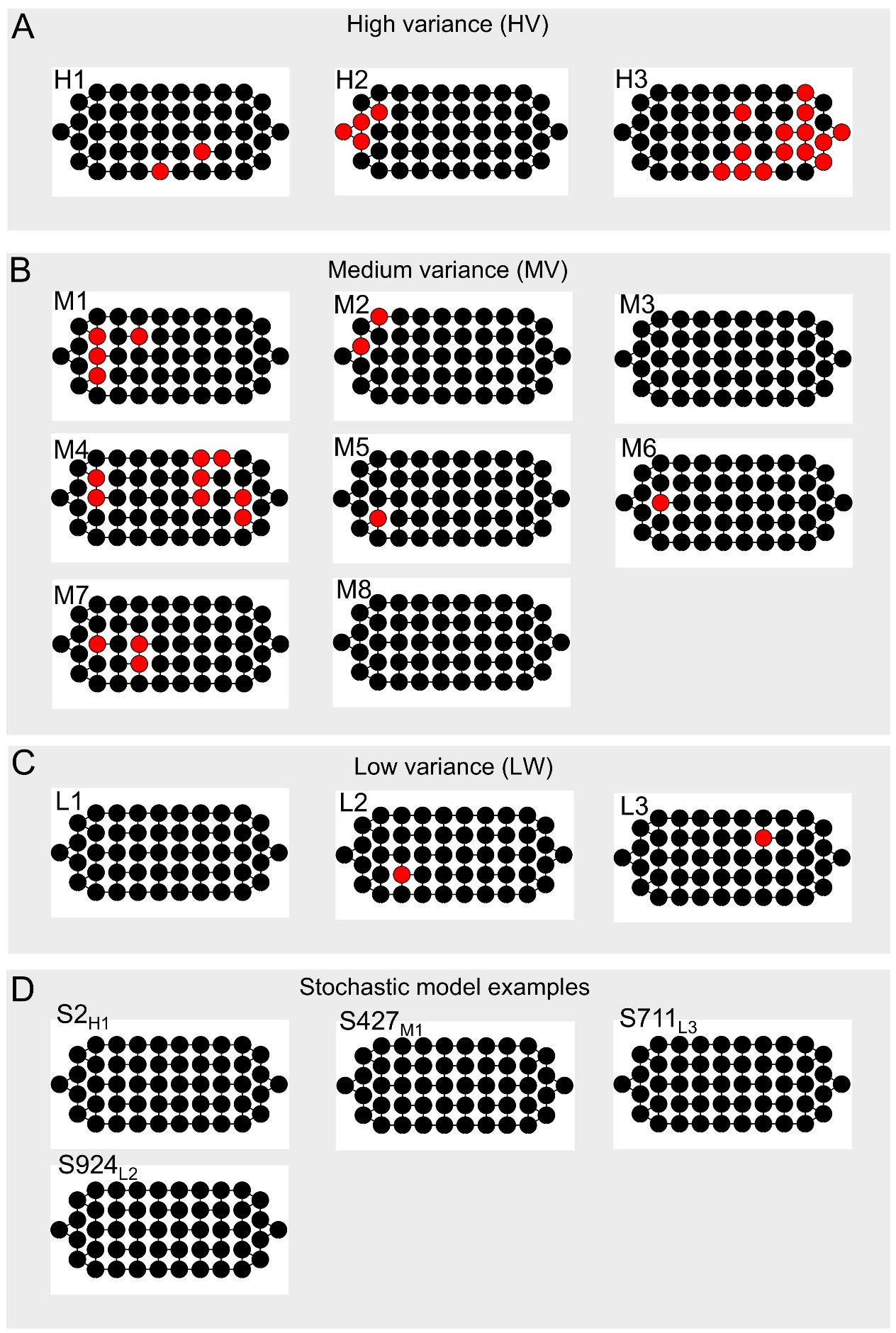


**Fig. S6. Chaos classification based on the Jacobian Lyapunov Exponent (LE) method across experimental and model data.** The schematic representation of the 50 patches on the chip highlights the timeseries which were classified as chaotic (red) and non-chaotic (black). **A**, High-variance experiments (n = 3). **B**, Medium-variance experiments (n = 8). **C**, Low-variance experiments (n = 3). **D**, Random selection of four model simulations with different initial abundances corresponding to one of the experiments as indicated by the name.


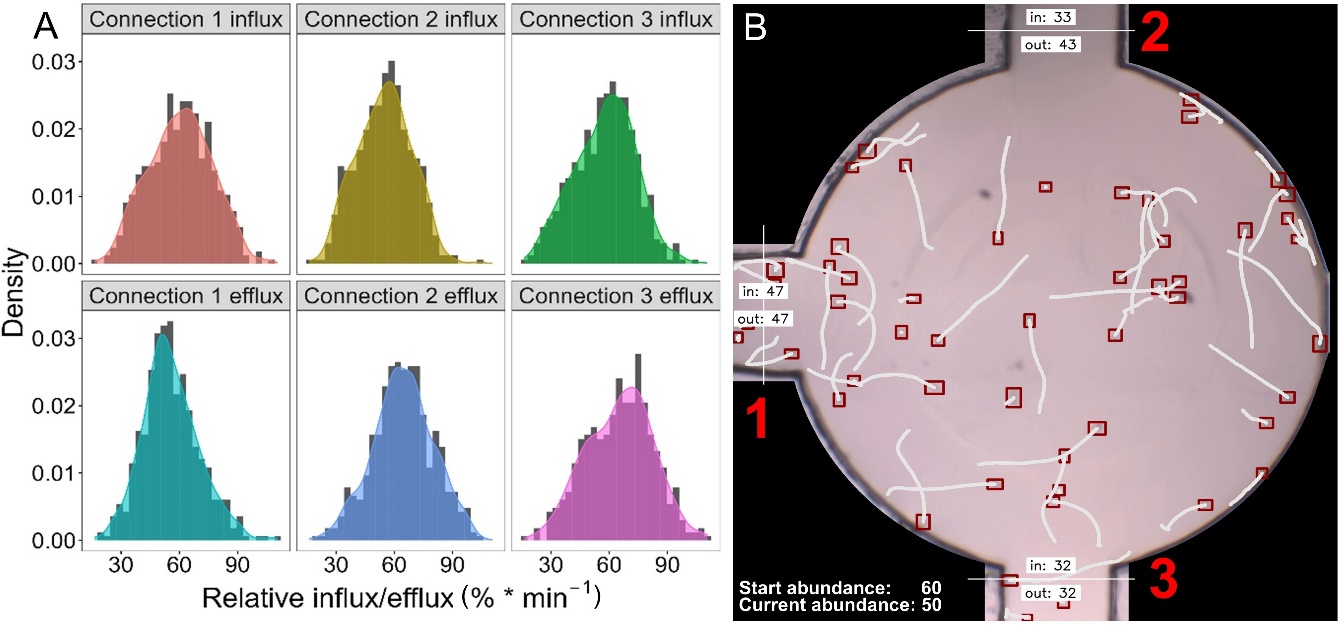


**Fig. S7 Calibration experiment for the estimation of model parameters**. **A**, Density plots of the relative influx/efflux per minute (transition rates) of *Tetrahymena* across all three connections of a patch (chamber), measured at two-minute intervals. Data were obtained from patch displayed in **B,** each set consisting of 510 observations. Each density curve approximates a normal distribution, with parameters $\mu= 0.6$ and $\sigma= 0.15$, reflecting the values used in the stochastical model. **B**, Representative example of the automated analysis (patch 39, approx. 5.5 hours after start of the experiment). Cells crossing the lines at the chamber connections are automatically counted as they enter or exit the chamber.
